# Association of vitamin D nutrition with neuro-developmental outcome of infants of slums in Bangladesh

**DOI:** 10.1371/journal.pone.0221805

**Published:** 2019-09-26

**Authors:** Fahmida Tofail, M. Munirul Islam, Mustafa Mahfuz, Md. Ashraful Alam, Shirina Aktar, Rashidul Haque, Md Iqbal Hossain, Dinesh Mondal, William A. Petri, Tahmeed Ahmed

**Affiliations:** 1 Nutrition and Clinical Services Division, International Centre for Diarrhoeal Disease Research, Bangladesh (icddr,b), Dhaka, Bangladesh; 2 Emerging Infections and Parasitology Laboratory, Infectious Diseases Division, International Centre for Diarrhoeal Disease Research, Bangladesh, Dhaka, Bangladesh; 3 Division of Infectious Diseases and International Health, Department of Medicine, University of Virginia, Charlottesville, Virginia, United States of America; Institut de Recherche pour le Developpement, FRANCE

## Abstract

The association of vitamin D with neuro-behavioral outcomes of young children is unclear, particularly of those who reside in tropical countries and are otherwise exposed to adequate sun light. To investigate this association, we analysed the existing data of poor infants, who participated in an observational, prospective-cohort (MalED) study, conducted in a slum of Dhaka-city. We assessed 265 infants aged 6–8 months for cognitive, motor, language and behavior development using Bayley-III. Information about infants’ temperament and communicative skills were provided by the mothers through a culturally modified “temperament-scale” and a “communicative-developmental inventory”. Serum concentration of vitamin D [25(OH)D] was measured in 205 infants. Around 28.3% of infants in this community had low level vitamin D, with the cut-off at <50 nmol/L. After controlling for all possible covariates, a multivariable-adjusted linear regression showed that children with vitamin D levels <50 nmol/L had significantly lower scores in two dimensions of temperament: activity (B±SE 1.64±0.78; 95%CI 0.10, 3.18; p = 0.037; effect size 0.37 SDs) and soothabilty (2.02±0.70; 0.64, 3.41; p = 0.004; 0.53 SDs), compared to children with vitamin D levels of ≥50nmol/L. These infants also scored low in word comprehensions (1.28±0.62; 0.05, 2.51; p = 0.042; 0.23 SDs) and were less active during test-procedures (0.33±0.16; 0.02, 0.64; p = 0.035; 0.27 SDs). Both the groups tested similarly in cognitive and motor scores. This study found, despite adequate sunlight-exposure, one in four infants of this slum-community are suffering from a subclinical vitamin D deficiency. Higher levels of vitamin D in these infants showed a positive association with temperament, language and behavior but not with cognitive and motor development. Our findings highlight the early-detected extra-skeletal neuro-behavioral role of vitamin D. Future studies in this area will give more insight.

## Introduction

Vitamin D is a fat-soluble vitamin and has a well-established role in bone health by maintaining calcium homeostasis [1]. Besides skeletal systems, normal level of serum 25-hydroxy vitamin D [25(OH)D] is necessary for maintaining immune, reproductive, epithelial, cardiovascular, nervous and muscular systems [2]. In humans, vitamin D is naturally synthesized in the skin under exposure to sunlight (Ultraviolet B). Dietary sources of Vitamin D are limited and can be obtained from fatty fish, fish oil, egg yolk, margarines and other fortified food products [3].

Global review of vitamin D status showed that in most cases intake of vitamin D is not optimum, particularly in countries where there is a scarcity of fortified food [4, 5]. One of the major active circulating metabolites of vitamin D is 25-hydroxy vitamin D [25(OH)D] that best reflects its clinical status. Synthesis of vitamin D in the skin from sun exposure also depends on different environmental factors such as geographic locations, weather, seasons, clouding and also individual factors such as the covering of the skin, anti-UV protection, skin pigmentation, age, and exposure to sunlight [6].

Deficiency of vitamin D is associated with various bone and non-bone health hazards [3]. Among non-bone health hazards, in recent decades, growing evidence identified association of low serum vitamin D levels with neuro-degenerative disorders, poor cognition, attention deficit hyperactivity disorder, autism, depression and schizophrenia in adolescents and adults [6–12].

The role of vitamin D in the brain is not well understood. However, there is evidence that vitamin D has neuro-protective functions [6, 13] and plays an important role in regulating the development, differentiation and ramification of nerve cells through influencing neurotrophic factors [6]. A meta-analysis on the brain MRI found association of brain cell atrophy with vitamin D depletion [14]. Receptors of vitamin D and the presence of specific enzymes for its conversion in active form has been identified in different parts of the brain particularly in prefrontal cortex, hippocampus, thalamus, hypothalamus, dopaminergic neurons and substantia niagra [15].

Data on neuro-developmental outcomes and vitamin D deficiency in children is inconsistent and controversial. Cut-offs for vitamin D deficiency, sufficiency and toxicity are based mainly on skeletal health outcomes [16, 17], which can be different for neuro-developmental outcomes.

There are scarcities of information about association of vitamin D levels with developmental outcomes of young children in tropical countries, who are otherwise exposed to adequate sun light.

To answer these questions, we explored the data of the Mal-ED cohort study that measured serum vitamin D levels and multiple neuro-developmental outcomes of young children. We investigate the association of serum vitamin D levels with cognitive, motor, language, temperament and behavioral development during infancy.

## Materials and methods

### Study subject and data collection

This exploratory study is a part of an ongoing longitudinal cohort study that is following 265 children from birth. The population is residing in poor slums of Dhaka City where most of the women are weavers and their husbands are generally day laborers or rickshaw pullers. Overcrowding, poor house construction and poor sanitary disposals are common features of the community. All the infants were assessed for developmental measures at the age of 6 to 8 months. However, vitamin D measures were available for 205 children around the age of 7 months.

### Developmental assessment

These measures included-

#### a) Global Intelligence

Assessment of children’s cognitive, motor, language and social-emotional development using Bayley Scale of Infant and Toddler Development, 3^rd^ edition (Bayley III). The Bayley III has not been standardized for Bangladeshi children but is being considered as the most widely accepted scale for measuring global intelligence of young children at individual level. We culturally adapted Bayley for Bangladesh and used it in several studies in rural [18–20] and urban [21] settings. As the test is mostly non-verbal, for cultural adaptation we mainly focused on some picture changes in the booklets by maintaining the original intent of the questions following standard procedures. We field tested the scale prior to the data collection of the main study, which was theoretically sensible. Short term test-retest reliability on 10 children (within 10 days gap) was also impressive (r > 0.75 for cognitive, motor and receptive language subscales). Two female testers, graduates in psychology or child development, unaware of the children’s group or study design, assessed the children in the presence of their mothers in a clinic setting. Inter-observer reliabilities (intra-class correlations) between testers and trainers were high, r = 0.99, p-value = 0.001 for all the three components. All the raw scores for cognitive, language, and motor development were converted to norm-referenced standardized scores (mean = 100, SD = 15) for composite scales. Ten percent of all tests (n = 25) were observed by the supervisors throughout the study period for ongoing reliability and video-tapes were checked for quality assurance.

#### b) Quality of home stimulation

Caldwell’s (1967) “Home observation of measuring environment (HOME)” inventory was used to assess the quality of stimulations available at home [22]. This scale was modified for use in Bangladesh and has also been used in our previous studies [23, 24]. The content of the original inventory remained the same and covered six domains of psychosocial stimulation (Organization of physical and temporal environment, stimulation, maternal involvement, play materials, avoidance/restriction /punishment and emotional and verbal responsiveness of the mother). One female interviewer (university graduate) visited all the homes and collected information from each mother in the presence of her baby. Before beginning the study, the inter-observer reliability between the trainer and the testers was high.

#### c) Temperament questionnaire

Maternal reports on infant temperament were collected using a culturally adapted questionnaire, derived from an existing validated instruments that was modified by Wach’s (Theodore Wachs, Purdue University, personal communication) for developing countries [25]. We asked 47 questions to the mothers from six subscales on activity, attention, positive emotionality, negative emotionality, how easy it is to soothe the child (soothability) and social approach (sociability) in Mal-ED study [26].

#### d) Communicative Development Inventories

To assess the language development of the children, we used an inventory that was based on the principles of the MacArthur Communicative Development Inventories: Words and Gestures. The inventory was previously validated for Bangladesh [27] and used in other developing countries [28]. In this study it collected information from the mothers about their infants’ ability to understand and/or say specific words

#### e) Serum vitamin 25(OH)D level

It was measured using an enzyme linked immunosorbent assay and the results were available for 205 infants aged 6–7 months. We are reporting about the outcomes of 205 infants who have both—vitamin D levels and developmental outcome measurements.

### Socio-economic status

We collected detailed information on housing, sanitation, parents’ income, education and family possessions, and the number of family members. There after we constituted different indices like, housing index (construction materials used for the roof, walls and floor of the house), crowding index (number of people per room), utility (quality latrine, sources of water and availability of electricity), and asset index (number of household possessions). Parental level of education and family income was used as a continuous variable [24].

### Anthropometry

Mothers’ height and weight were measured during enrolment. Infants’ weight, supine length and head circumference were measured around 7 months of age. All measurements were made using standard techniques [29]. Weight and length measurements were converted to weight-for-age (WAZ), length-for-age (LAZ) and weight-for-length (WLZ) Z-scores according to the new WHO child growth standards [30]. Maternal BMI was also computed.

### Statistical analysis

We entered and analyzed data using SPSS for WINDOWS (version 11.5; SPSS Inc, Chicago) and *p*-values < 0.05 were considered statically significant. Distribution of each variable was checked for normality, and where necessary appropriate transformations or categorizations were made. Vitamin D distribution and all developmental outcome variables (cognitive, language, motor, temperament subscales and behavior subscales) were normally distributed except soothability and attention subscales of Infant Temperament Scale. These two subscales were slightly skewed (positively) and log transformed for normality before using in correlation matrices and regression analysis. Estimates with or without log transformation was almost similar, so for better understanding we presented the mean values of untransformed soothability and attention scores in the table of group comparison. We first analyzed whether exposure (serum vitamin D level) measure was associated with outcome variables (developmental measures) around a child’s 7 months of age. Then we tested the crude associations between exposure, developmental outcomes, and potential confounders (socio-demographic and biological) using Spearman correlation coefficient. Based on cut-off level for vitamin-D sufficiency among infants and children [16], we categorized distribution of vitamin D to deficient (< 50nmol/L) and sufficient (≥ 50nmol/L) groups to compare the group-differences of all developmental outcomes, using independent sample t-test. Finally, in the multiple linear regression analyses, we adjusted for the variables that were significantly correlated with both exposure or outcome variables. We found strong correlation between birth-weight and height for age Z scores (r = 0.88) around 7 months. So, to avoid multicollinearity, we selected height for age Z scores as it was having stronger association with developmental outcomes compared to birth weight. So, in the final regression model we adjusted for all variables those were associated with either exposure or outcomes (age of the child during assessment, asset score, maternal education, amount of stimulation received at home, height for age Z score at the time developmental assessments and head circumference at 7 months).

### Ethics

The research and ethical review committees of icddr,b approved the study protocol and prior to that informed consents were obtained from the parents of the children.

## Results

In general, the study population was from low income community and about 23% of the families had monthly income equal or less than USD$ 70. Table 1 represents the baseline characteristics of the population. Around 63% of the families lived-in poor quality homes with single-room and shared latrines, where18% latrines were pit latrine without flush. Half of these families had more than four family members and a third of the parents completed education beyond primary level.

**Table 1:** Baseline characteristics of the study population (n = 205).

Socio-demographic information	Mean ± SD or %
Monthly income ≤USD 70	23%
House with 1 room	63%
Family size (>4 members)	50%
Asset Index (range 1–13, higher is better)	6.9±2.5
Crowding Index (# of people/room)	3.6±1.2
Housing Index (range 13–23, higher is better)	19.0±1.8
Utility Index (4–9, higher is better)	7.0±1.4
Total HOME score	33.7±4.1
Mothers with schooling >5 years	34.1%
Fathers with schooling >5 years	27.8%
Water source (piped water in the yard)	83%
**Maternal information**	
Maternal age in year	25 ± 5
Parity one	32%
Maternal body mass index (BMI)	22.2±3.5
**Child information**	
Birth weight (kg)	2.8 ± 0.4
Age in months during assessments	6.4±1.3
Low birth weight <2.5 kg	25%
Head circumference in cm at 7 months	41.8±1.4
Height- for- age z score between 7–8 months	-1.3±0.95
Girl child	52%

Values are mean ± SD or %

HOME = Home observation of measuring environment; cm = Centimeter

Mean maternal BMI was 22.2±3.5 and a quarter of the newborns were low birthweight (birth weight < 2.5 kg). Only 13% infants of this population had exclusive breastfeeding up to six months (national recommendation). At the age around 7 months, 21% infants were stunted and 14% were wasted (Z scores ≤-2). Mean height for age Z score among the children was -1.3±0.97.

Bivariate correlation showed that age of the child during assessment, birth weight, mothers’ education, asset score, amount of stimulation received at home, head size at 7 months and height for age Z score around 7 months are positively associated most of the developmental outcomes Table 2.

**Table 2:** Correlation of socio-demographic variables with Vitamin D level and outcome variable^1^.

N = 205	Cognitive composite score	Language composite score	Social emotional composite score	Motor composite score	Language expression	Language compre-hension	Sociability score	Sooth-ability score*	Activity*	Attention	Positive emotion
Vitamin D nmol/L	-0.03^0.63^	-0.07^0.33^	0.01^0.85^	-0.04^0.60^	-0.05^0.51^	0.07^0.36^	0.16^0.025^	0.23^0.001^	0.17^0.015^	0.04^0.62^	0.07^0.32^
ASSET Index	0.09^0.21^	0.10^0.14^	0.15^0.04^	0.14 ^0.04^	0.12^0.10^	0.16 ^0.021^	-0.07^0.33^	-0.14^0.04^	-0.06^0.39^	-0.15^0.03^	-0.02^0.76^
Housing Index	0.08^0.27^	-0.003^0.97^	-0.10^0.15^	0.05^0.51^	0.07^0.35^	-0.02^0.79^	0.00	0.05^0.49^	-0.004^0.95^	-0.01^0.88^	0.06^0.37^
Utility Index	0.11^0.14^	0.02^0.77^	-0.08^0.27^	0.06^0.39^	-0.04^0.54^	-0.02^0.83^	0.09^0.19^	0.02^0.77^	-0.13^0.08^	-0.03^0.66^	0.03^0.63^
Mothers’ education (years in school)	0.10^0.15^	0.10^0.14^	0.23^0.001^	0.10^0.18^	0.11^0.11^	0.15^0.04^	-0.03^0.64^	-0.006^0.93^	-0.04^0.59^	-0.10^0.14^	-0.006^0.94^
Fathers’ education (years in school)	-0.05^0.58^	0.09^0.31^	0.06^0.51^	-0.09^0.29^	-0.02^0.87^	0.05^0.55^	0.15^0.03^	-0.03^0.77^	-0.11^0.20^	-0.13^0.16^	0.04^0.70^
HOME score	-0.03^0.72^	0.07^0.35^	0.15^0.03^	0.02^0.82^	0.10^0.15^	0.20^0.005^	-0.30^<0.001^	-0.34^<0.001^	-0.19^0.006^	-0.39^<0.001^	-0.32^<0.001^
Mothers’ age in years	0.03^0.66^	0.02^0.82^	-0.17^0.02^	0.06^0.41^	-0.19^0.01^	-0.05^0.49^	0.11^0.12^	0.07^0.34^	0.06^0.40^	0.09^0.21^	0.04^0.55^
Birth-weight (kg)	0.20 ^0.005^	0.10^0.15^	0.01^0.92^	0.26^<0.001^	0.16^0.03^	0.09^0.22^	0.08^0.24^	0.04^0.62^	0.04^0.55^	0.007^0.93^	-0.02^0.79^
Child’s age in months during test	0.02^0.76^	-0.11^0.13^	0.06^0.37^	-0.06^0.38^	-0.008^0.92^	0.03^0.67^	-0.10^0.15^	-0.07^0.32^	-0.05^0.46^	-0.15^0.04^	-0.08^0.23^
height- for- age Z score around 7-months	0.15^0.04^	0.005^0.94^	0.03^0.64^	0.22^0.001^	0.08^0.24^	0.07^0.32^	0.14^0.05^	0.05^0.46^	0.15^0.04^	-0.03^0.72^	0.005^0.94^
Head size around 7 months	0.18^0.01^	0.03^0.71^	0.01^0.88^	0.05^0.49^	0.15^0.04^	0.11^0.12^	0.13^0.06^	0.13^0.07^	0.06^0.42^	0.05^0.48^	0.10^0.15^

Bi-variate (Spearman’s) correlation, HOME = Home observation of measuring environment; BMI = Body mass index

^1^Values are in correlation co-efficient with p-values in superscript

*Values are log_10_ transformed for mild positive skeweness

Table 2 also presents significantly association of serum vitamin D [25(OH)D] level was with three out of six subscales of Infant Temperament Scale: activity (r = 0.17, p = 0.015), sociability (r = 0.16, p = 0.025) and soothability (r = 0.23, p = 0.001). Higher vitamin D level was also associated with some better behavioral outcomes of the infants that almost approached significance i.e., they were more active (r = 0.13, p = 0.056) and they vocalized more (r = 0.12, p = 0.087) during test administration compared to the infants with lower vitamin D level. Other developmental outcomes showed no correlation with vitamin D level in this population.

**Fig 1:**
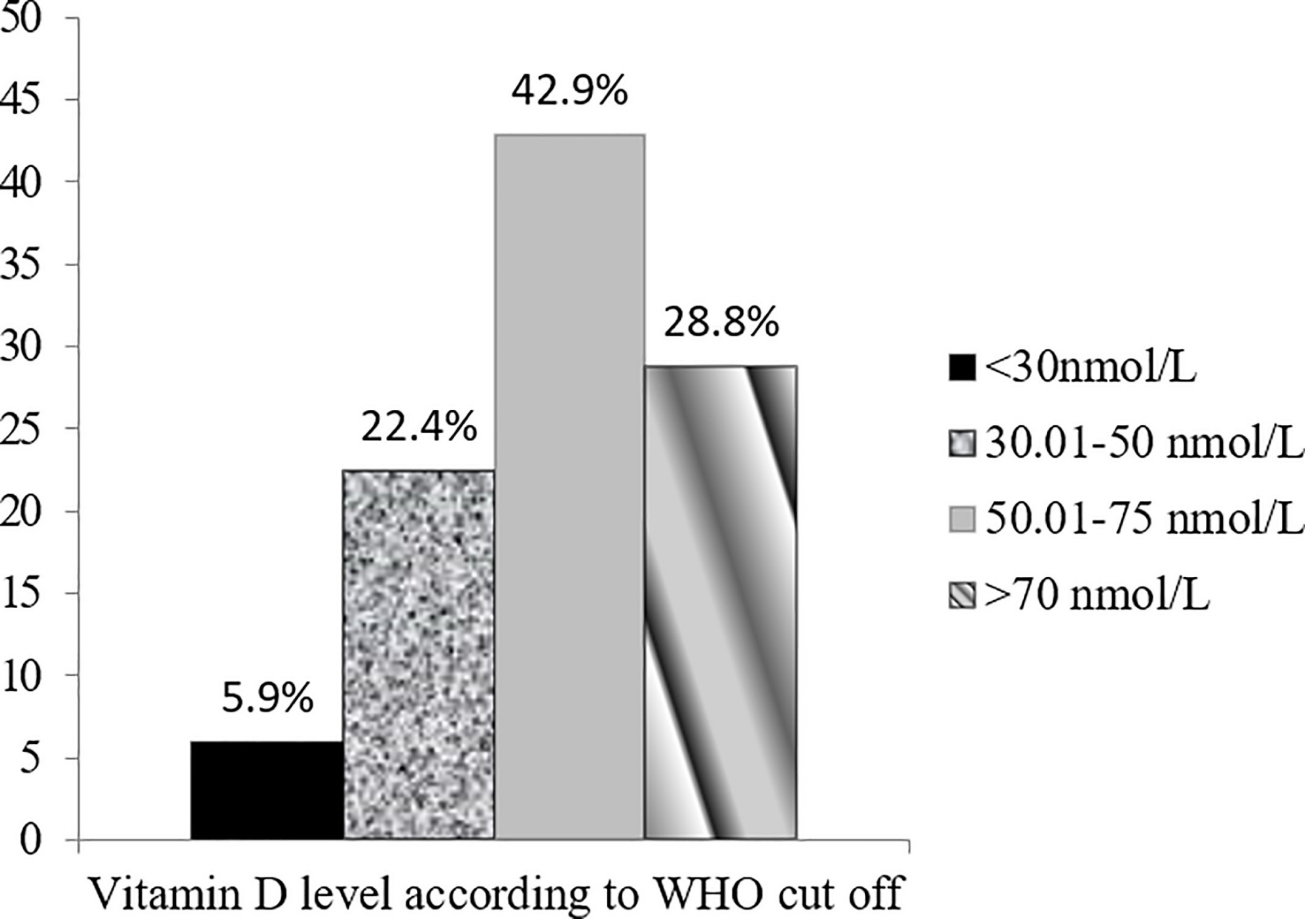
Distribution of vitamin D level in the population.

Of the 205 children aged around 6 months, the mean±SD of vitamin D level was 64.5 ±23 nmol/L (95% CI 61.3–67.7) and the median value was 63.8 (IQR 48.3, 78.9). In this population the highest vitamin D level was 148.6 nmol/L and the lowest was 8.4 nmol/L. Around 4.6% children had vitamin D level >100 nmol/L and 2.9% had <25 nmol/L. Fig 1 displays distribution of vitamin D in the population based on some commonly used cut-offs.

As there is no universal definition for cutoff value of vitamin D deficiency in infants and children, particularly for non-bone health outcomes, we considered the cutoff value <50 nmol/L for vitamin D deficiency and ≥50 nmol/L sufficiency based on vitamin D review and recommendations for children [16]. We found 28.3% children were vitamin D deficient (<50 nmol/L) and in group comparison they scored significantly lower in two (activity and soothability) out of six temperament subscales compared to vitamin-D adequate (≥50nmol/L) children. They also displayed trend of lower score in one temperament subscale (sociability) and one behavior subscale (activity) of children that reached near to significance. There was no difference in cognition, language and motor outcomes between groups (Table 3).

**Table 3:** Distribution of cognitive, motor, language, temperament and behavioral scores of children by vitamin D deficient (< 50 nmol/L) and sufficient (≥ 50 nmol/L) group (unadjusted comparison).

	Vitamin D level * < 50 nmol/L	Vitamin D level * ≥ 50 nmol/L	Group Difference p value (95% CI)
	n = 58	n = 147	
Cognitive composite score ^1^	100.7±11.7	100.2±10.5	0.76 (-2.8 to 3.8)
Motor composite score ^1^	99.3±12.7	97.8±12.4	0.47 (-2.4 to 5.2)
Language composite score ^1^	93.7±11.2	91.7±11.2	0.26 (-1.5 to 5.4)
Social emotional composite score ^2^	88.7±13.4	88.0±15.3	0.77 (-3.8 to 5.2)
Expressive language ^2^	2.3±1.1	2.3±1.0	0.76 (-0.3 to 0.4)
Comprehensive language^2^	8.5±3.8	9.4±3.9	0.18 (-2.0 to 0.4)
Temperament–Activity^2^	13.6±5.2	15.4±4.8	0.02 (-3.4 to -0.3)
Temperament–Attention ^2^	13.7±4.0	13.9±4.8	0.77 (-1.6 to 1.2)
Temperament–Negative Emotionality^2^	28.6±5.0	27.8±6.0	0.38 (-1.0 to 2.6)
Temperament–Positive Emotionality ^2^	13.7±3.9	14.6±3.9	0.17 (-2.0 to 0.4)
Temperament- Sociability ^2^	9.4±4.5	10.5±4.1	0.09 (-2.4 to 0.2)
Temperament- Soothability ^2^	10.37±4.2	12.76±4.8	0.001(-3.8 to -1.0)
Approach of the child during test ^3^	4.7±0.7	4.7±0.7	0.88 (-0.2 to 0.2)
Activity of the child during test ^3^	5.0±1.0	5.2±1.0	0.07 (-0.6 to 0.02)
Emotion of the child during test^3^	5.2±0.7	5.2±0.9	0.57 (-0.3 to 0.2)
Cooperativeness of the child during test^3^	4.7±0.7	4.8±0.8	0.95 (-0.3 to 0.2)
Vocalization of the child during test ^3^	2.5±1.2	2.8±1.4	0.16 (-0.7 to 0.1)

Independent sample t test

*mean values of untransformed developmental outcome data

^1^Direct measurement on infants

^2^Mother’s report

^3^Observation during test

Finally, multivariable-adjusted linear regression (Table 4) was conducted by controlling for all possible covariates that are associated with either serum vitamin D level or developmental outcomes (mentioned above). Results showed that vitamin-D deficient children had significantly lower scores in activity (B±SE 1.64±0.78; 95%CI 0.10, 3.18; p = 0.037), and soothability (2.02±0.70; 0.64, 3.41; p = 0.004) subsacles of temperament according to mothers’ report compared to vitamin D adequate children. These children also understood lesser number of words (1.28±0.62; 0.05, 2.51; p = 0.042) as per mothers’ report and observed to be less active (0.33±0.16; 0.02, 0.64; p = 0.035) during Bayley’s developmental assessments. However, there was no difference between the groups on cognitive, motor or language scores when assessed directly using Bayley Scale of Infant Development-III.

**Table 4:** Multivariable-adjusted linear regression analysis showing infants’ developmental outcome scores by vitamin D deficient (<50 nmol/) and sufficient (≥50 nmol/) groups.

Developmental Tests		B ±SE (95% CI)	P value
	Subtests		
Bayley	- Cognitive composite score	-0.84±1.71 (-4.21, 2.52)	0.62
	- Motor composite score	-0.65±1.97 (-4.54, 3.25)	0.74
	- Language composite score	-2.32±1.82 (-5.92, 1.27)	0.20
	- Socio-emotional composite score	-0.29±2.32(-4.88, 4.29)	0.90
Temperament	- Activity	1.64±0.78 (0.10, 3.18)	0.037
	- Positive emotion	0.51±0.58 (-0.64, 1.66)	0.38
	- Negative emotion	-0.75±0.94 (-2.61, 1.11)	0.43
	- Soothability	2.02±0.70(0. 64, 3.41)	0.004
	- Soothability (log transformed)	0.03±0.01(0. 008, 0.05)	0.007
	- Sociability	0.85±0.63 (-0.38, 2.09)	0.18
	- Attention	-0.35±0.66 (-1.65,0.96)	0.60
	- Attention (log transformed)	-0.02±0.02 (-0.06,0.02)	0.39
CDI	- Language comprehension	1.28±0.62 (0.05, 2.51)	0.042
	- Language expression	-0.02±0.17 (-0.32,0.36)	0.91
Behavior	- Approach during test	0.02±0.12 (-0.21, 0.25)	0.86
	- Activity during test	0.33±0.16 (0.02, 0.64)	0.035
	- Emotional tone during test	0.10±0.14(-0.17, 0.38)	0.45
	- Cooperativeness during test	0.06±0.12 (-0.18, 0.30)	0.63
	- Vocalization during test	0.28± 0.21 (-0.14, 0.70)	0.19

Intervention effect B is the regression coefficient and SE is the Standard Error. Ranges shown are 95% CIs. Data were assessed in 58 infants in the vitamin D deficient (<50 nmol/L = 1) group versus 147 children in the vitamin D sufficient (≥50nmol = 2) group; these results were adjusted for the age of the child during assessment, asset score, maternal education, amount of stimulation received at home, height for age Z score (measured at the time of test between 7–8 months) and head circumference at 7 months

The effect sizes of these differences (adjusted means/pooled SD) for activity and soothability subscales of temperament were impressive, 0.37 and 0.53 SDs respectively. Effect size for both- language comprehension and activity during test were relatively small, 0.23 and 0.27 SDs respectively.

## Discussion

In our study, vitamin D deficiency [serum 25(OH)D concentration <50nmol/L] during infancy showed differences in some traits of reported temperament, reported receptive language, and directly observed behavior. These differences were significant when adjusted for all possible covariates e.g. age of the child, mothers’ education, asset/wealth, stimulation received at home, head circumference and height for age Z score. Mothers also reported lower ability to comprehend the language among these children. We did not find any association of vitamin D status of infants with their cognitive or motor scores. Vitamin D status also did not differ between boys and girls or between stunted and non-stunted children (HAZ > or ≤ -2.00).

In this study, it is interesting to see the association of vitamin D with infants’ two (activity and soothability) out of five measured temperament traits (activity, soothability negative emotion, positive emotion and sociability). According to the mothers report, vitamin D deficient (<50nmol/L) children were reported to have lower activities and were ‘difficult to soothe’, which means difficulties in reducing their fuss, cry, irritation or distress when soothing techniques are applied by the caretakers, compared to vitamin D sufficient (≥50nmol/L) infants around 7 months.

Temperament can be defined as individual differences in emotional, motor and attentional reactivity and self-regulation [31]. It covers the dimensions of behavioral traits that are biological or constitutional, present at birth, relatively consistent across settings. It remains stable over time and can be modified by interaction of heredity, life experience, environment and maturation. Wachs (2009) reported that it can be influenced by nutrition during infancy as well. Temperament during infancy is important as its certain pattern is reported to be related to an increased risk of later behavior problems [32]. We could not locate any study that looked for association between vitamin D status and traits of temperament in young children, but there are studies on older children and adults, showed its inconsistent association with behavioral problems. One longitudinal study on school aged children showed weak association of serum concentration of vitamin D [lower 25(OH)D_3_] with more prosocial behavioral problems at around 12 years of age in adjusted model [33]. This study also showed the association of a lower vitamin D level with depressive symptoms and academic performances at the age of around 14 years [34, 35]. Two studies assessed maternal serum 25(OH)D concentration during pregnancy and found no association with offspring’s later behavioural development in childhood [36, 37]. In contrast one study on adults showed significant association of higher vitamin D metabolite [1,25 (OH)_2_D_3_] with fewer behavior problems reflecting more extrovert and open behavior [38]. It is possible that in our study, vitamin D deficient infants with lower activity and soothability traits of temperament, may become introvert and emotionally vulnerable in future. Among all measured traits of temperament scale, soothability specifically reflects threshold, intensity, adaptability of response and easy distractibility of a child that has relatively high stability over time to explain later behavior traits. In consistence with Thomas et al. (1968) [39] we also found positive correlation of soothability with almost all the other temperament traits e.g. activity (r = 0.5, p <0.001), attention (r = 0.4, p <0.001), positive emotionality (r = 0.4, p <0.001) and sociability (r = 0.3, p <0.001) that were measured, indicating an internal consistency of the measurement. As temperament in general reflects a personality trait in adulthood, our findings align with findings of Ubbenhorst and colleagues (2011) where they found association of concurrent vitamin D status with a personality trait in adults [38]. However, due to differences among age-ranges of study-population, geographic locations, assessment tools, and cut off values of vitamin D deficiency for non-bone health, it is difficult to compare our findings with other study findings conclusively.

We also found a lower ability of vitamin D deficient infants to comprehend the language compared to vitamin D sufficient infants. Although we could locate no other study that looked for this association during infancy, two studies showed significant liner association of vitamin D deficiency during mid [36] or late pregnancy [40] with later language development of the offspring. They explained the possible reasons could be the interference in the development of the Perisylvian structures in the brain during neurogenesis due to vitamin D deficiency, which are responsible for language in children. However, both the studies considered a cut off of maternal vitamin D deficiency ≤46nmol/L [36] and <37.5 nmol/L [40]. We are unaware of any established cut-off for vitamin D level during pregnancy that might play a role on neuronal architecture of language apparatus of the developing brain of the foetus. Our children were very young, around 7 months of age and the reports on language comprehension was obtained from mothers to see the association of the concurrent vitamin D status of these children. Recent research also suggests that vitamin D deficiency during early childhood may be an environmental trigger for autism spectrum disorder in infants that indicates a problem in communication, who are possibly genetically predisposed to autism [7,41, 42]. Our findings highlight the importance of future research in this area.

When we directly observed for five behaviors (approach, activity, emotion, co-operation and vocalization) of these vitamin D deficient children during Bayley test, we only found them to be less active according to a 9-point rating scale. This finding supports the mothers report about the lower activity level of these children during temperament assessment. We do not know whether less active behavior in these vitamin D deficient children are mediating through their bone health.

However, the accurate role of vitamin D deficiency on neuro-developmental and behavioral outcomes is not clear. We also do not know for how long these children are vit-D deficient. Studies in vivo and vitro indicates possible role of vitamin D on neurodevelopment through Ca(2+) signalling, antioxidant function, metabolic regulation of neurotrophins and neurotoxins, protecting the brain from inflammation [43–46].

In this age group, we did not find any effect of vitamin D deficiency on cognitive and motor function, although positive associations were reported in elderly and middle-aged people [47–50]. It is possible that cognitive features may become apparent in later life.

Assessing cognitive development at early age is complex and difficult as motor developments predominate during that phase. We assessed cognition and motor development using Bayley Scale (Bayley-III), which is although considered as Gold Standard of developmental measurement, predicts early cognition poorly in under-two children [51]. This finding is consistent with the reports of vitamin D that looked for its association with cognition or IQ of children or adolescents [52–54]. Recent reviews in adults also reported about the inconsistent role of vitamin D on cognition [55].

We had a number of limitations in this exploratory study as it is a part of an ongoing cohort study. This finding is based on only a one-time point assessment of vitamin D, we are not aware of maternal pregnancy vitamin D levels or measures for sunlight exposure of these infants. We also do not have any dietary details of these children that contained vitamin D. However, almost 97% of these infants started breastfeeding soon after birth within a week and the vitamin D status did not vary according to their nutritional status. Our major strength is we had a number of developmental assessment measures and those correlated with each-other in a meaningful way indicating concurrent validity of the assessments. We also became able to control a number of important variables that commonly affects developmental outcomes e.g. home environment, maternal education and nutritional status of the children. For being a part of a longitudinal cohort, we will be able to follow these children for their future development.

## Conclusions

According to our knowledge this is the first report that is showing a possible association of vitamin D with temperament, behavior and language development at a very young age when brain growth spurt occurs to promote early learning. Despite an adequate sun exposure one out of four infants living in these slum areas are suffering from subclinical vitamin D (<50mmol/L) deficiency. Due to poor socio-economic status of this population, we assume that they were possibly deficient in dietary vitamin D as well. Follow-up of these children will give more insight about the consequences of this relationship in early childhood. Our findings highlight the importance of a more well-designed future research in this area for developing comprehensive intervention at an early age to promote better neuro-cognitive development and behavior.

## Supporting information

S1 Dataset Vit D & temperament. (SAV)
